# Epigenetics of Aging and Aging-Associated Diseases

**DOI:** 10.3390/ijms22010401

**Published:** 2021-01-02

**Authors:** Dominik Saul, Robyn Laura Kosinsky

**Affiliations:** 1 Kogod Center on Aging and Division of Endocrinology, Mayo Clinic, 200 First St SW, Rochester, MN 55905, USA; Saul.Dominik@mayo.edu; 2 Department of Trauma, Orthopedics and Reconstructive Surgery, Georg-August-University of Goettingen, 37075 Goettingen, Germany; 3 Division of Gastroenterology and Hepatology, Mayo Clinic, 200 First St SW, Rochester, MN 55905, USA

**Keywords:** epigenetics, histones, histone modifications, aging, aging-associated diseases, diabetes, CDKN2A, osteoporosis, sarcopenia, gene expression

## Abstract

Aging represents the multifactorial decline in physiological function of every living organism. Over the past decades, several hallmarks of aging have been defined, including epigenetic deregulation. Indeed, multiple epigenetic events were found altered across different species during aging. Epigenetic changes directly contributing to aging and aging-related diseases include the accumulation of histone variants, changes in chromatin accessibility, loss of histones and heterochromatin, aberrant histone modifications, and deregulated expression/activity of miRNAs. As a consequence, cellular processes are affected, which results in the development or progression of several human pathologies, including cancer, diabetes, osteoporosis, and neurodegenerative disorders. In this review, we focus on epigenetic mechanisms underlying aging-related processes in various species and describe how these deregulations contribute to human diseases.

## 1. Introduction

Aging is a multifactorial biological process of declining physiological functions increasing the susceptibility to aging-related chronic diseases, such as cancer, metabolic, cardiovascular, musculoskeletal, as well as neurodegenerative diseases [1]. Numerous studies have focused on the decipherment of the hallmarks of aging in order to identify potential therapeutic targets to mitigate the aging process. Hallmarks of aging include stem cell exhaustion, altered intercellular communication, senescence, genomic instability, and epigenetic deregulation [2].

Epigenetics refers to reversible heritable mechanisms, which can affect gene expression without underlying changes in DNA sequences, but rather via chromatin modifications. Eukaryotic chromatin is a highly condensed structure containing repeating structural subunits, the nucleosomes. Each nucleosome consists of a histone octamer assembled of two copies of each histone (H2A, H2B, H3, and H4, as well as histone variants, such as macroH2A, H3.3 and H2A.Z), wrapped around by 147 base pairs of DNA [3,4]. Each core histone possesses histone-fold domains serving for the interaction of the histones and N-terminal histone-tails. These tails can be subjected to post-translational modifications, which frequently affect gene expression. These modifications include, for instance, histone acetylation, methylation, phosphorylation and ubiquitination [5].

Epigenetics is a rapidly evolving research field and there is a profound interest in therapies targeting epigenetic as well as aging-related processes. In this review, we focus on aging-associated epigenetic regulatory mechanisms and highlight their implications in aging-related diseases.

## 2. Epigenetics of Aging and Aging-Related Diseases

### 2.1. Epigenetic Changes in Aging

#### 2.1.1. Histone and Heterochromatin Loss

The DNA is organized into complex three-dimensional structures; however, for gene transcription, the DNA sequence has to be accessible to key regulators, such as transcription factors and RNA polymerases. Besides chromatin remodeling, which results in the rearrangement of chromatin structures, the global number of histones defines DNA accessibility [6]. In fact, the loss of histones during cellular aging is one of the key observations from simple eukaryotic models, including yeast, to mice and humans. In a micrococcal nuclease-DNA sequencing (MNase-seq) approach detecting protein-unbound DNA regions in young and old *Saccharomyces cerevisiae*, a nucleosome loss of approximately 50% was detected. As a consequence, global transcription levels were highly upregulated in aged cells [7]. Similarly, aging human fibroblasts grown in vitro showed a replication-associated reduction in histone biosynthesis and quiescent satellite cells displayed decreased histone expression [8,9].

Reduced synthesis of histones together with changes in chromatin structure (see Section 2.1.4 and Section 2.1.5) leads to a global loss of constitutive heterochromatin, one of the earliest models associated with aging. Heterochromatin loss, the transition from highly condensed to tightly packed chromatin structures, during aging has been observed across many species. As a consequence, modified chromatin architecture, the de-repression of silenced genes and global gene expression changes can occur [10].

#### 2.1.2. Histone Variants

Besides the loss of histones, the exchange of canonical histones (H2A, H2B, H3, and H4) with histone variants was observed in aging organisms. These histone variants display distinct primary sequence and properties compared to canonical histones, thereby regulating gene transcription programs. Various aging-related studies evaluating histone variants in murine, primate and human cells implicate a high enrichment of macroH2A (mH2A), H3.3 and H2A.Z. In general, the incorporation of histone variants into the chromatin can be replication-coupled or replication-independent. The replication-coupled process results in a genome-wide incorporation of new nucleosomes into gaps between pre-existing nucleosomes. In contrast, the replication-independent addition of nucleosomes or subunits occurs locally. Thus, during the replication-independent process, histone variants can replace canonical histones, thereby potentially altering gene expression programs [11,12]. The mH2A isoforms are characterized by the presence of a C-terminal 30 kDa non-histone macro domain [13,14], and were shown to facilitate the activation of transcription factors during differentiation processes [13] and the prevention of the reactivation of pluripotency-associated genes [15]. Notably, human fibroblasts undergoing replicative senescence in vitro as well as several tissues isolated from aging mice and primates displayed an enrichment in mH2A levels [16].

Another example is the H3 variant H3.3, which differs from the canonical form by only four amino acids. It was shown to be incorporated only in a replication-independent manner and to be enriched in transcriptionally active chromatin regions. Recent aging studies in mice revealed that H3.3 accumulates in various tissues during aging and that the canonical isoforms have been almost completely replaced by this histone variant by the age of 18 months [17]. Moreover, H3.3 was linked to aging processes in *Caenorhabditis elegans*. Here, the deletion of H3.3 resulted in profound transcription changes of longevity-associated genes and in decreased survival [18]. Similar results were found when analyzing postmortem human brains where H3.3 levels gradually increased over the first decade of life. In individuals who were 14 to 72 years old, H3.3 amounts remained stable [19].

Another well-characterized histone variant is H2A.Z, which only shares approximately 60% similarity with the canonical H2A form. There are numerous studies elucidating the function of H2A.Z; however, some findings are contradictory. For instance, H2A.Z was associated with transcriptional activation, transcriptional repression, cell cycle control, DNA replication and DNA damage repair [12]. A recent study uncovered a memory-suppressing function of H2A.Z in mice. While H2A.Z accumulated during aging, active learning resulted in H2A.Z reduction and learning-induced gene expression patterns [20].

A variant of H2AX, which is phosphorylated at the C terminal serine-139 by Ataxia-Telangiectasia-Mutated and Ataxia Telangiectasia and Rad3-related (ATM/ATR), appears during the response to double strand breaks (DSBs). Radiation induced phosphorylation of H2AX in short time ranges, referred to as γH2AX, can be used as biological dosimeter. Together with Senescence-Associated β-galactosidase (SA-β-gal) staining, γH2AX is frequently used to detect senescent cells, highlighting DSBs and telomere shortening [21,22,23].

#### 2.1.3. DNA Methylation

Besides histone methylation, DNA can be directly methylated through the covalent linkage of a methyl group to the fifth position of the cytosine ring to generate 5-methylcytosine (5mC). This modification is mainly present in DNA regions rich in cytosine-phospho-guanine (CpG) dinucleotides. While there is extensive evidence that DNA methylation at promoter regions is associated with gene silencing, the decipherment of the function of gene body methylation is still ongoing [24,25,26]. The repression of transcription due to covalent addition of methyl groups onto the DNA can be mediated by interfering with the site-specific binding of transcription factors or by the recruitment of methyl-CpG-binding domain proteins [27,28]. The transfer of this heritable epigenetic mark is mediated by DNA methyltransferases (DNMTs) including DNMT1, DNMT2, DNMT3A, DNMT3B, and DNMT3L. While DNMT1 has a maintenance function, the de novo establishment of DNA methylation is exerted by DNMT3A and DNMT3B alone or in a complex with DNMT3L [29,30].

The conversion of 5mC to the unmodified state is thought to be mediated in an “active”, enzyme-dependent or in a “passive” demethylation process. The family of Ten Eleven Translocation (TET) proteins, TET1, TET2, and TET3, are able to erase DNA methylation in an “active” stepwise process [31,32]. These factors catalyze the oxidation of 5-methylcytosine (5mC) to the intermediates 5-hydroxymethylcytosine (5hmC), 5-formylcytosine (5fC), and 5-carboxylcytosine (5caC). After the recognition of 5fC and 5caC by the Thymine DNA Glycosylase (TDG), the oxidized cytosine base is excised. Finally, this abasic site will be recognized and replaced by an unmodified cytosine residue by Base Excision Repair (BER). During “passive” DNA demethylation, 5-methylcytosine is diluted in a replication-dependent process during cell division [33,34]. Interestingly, it has been demonstrated that a high abundance of 5hmC represses DNMT1 activity by 60-fold, suggesting a role of TET-mediated induction of “passive” demethylation [35].

While methylation-associated control of gene expression pattern is essential for mammalian development and further cellular processes, it was thought to be dispensable in several organisms such as *Caenorhabditis elegans* and *Drosophila melanogaster* [36]. Recent studies describe the methylation of exocyclic NH2 groups at the sixth position of the purine ring in adenines (6 mA) in *C. elegans*, which is thought to be regulated through the DNA demethylase NMAD-1 and the DNA methyltransferase DAMT1 [37]. In addition, there is emerging knowledge on species- and life cycle-dependent 5mC levels among the genomes of the members of genus *Drosophila* [38].

While it has been known for several decades that DNA methylation can regulate gene expression patterns, biological consequences are still not fully investigated. Generally, it has been described that CpGs at promoter regions display hypermethylation while other CpGs undergo hypomethylation during aging [39]. Remarkably, two large-scale studies significantly contributed to the understanding of the relevance of DNA methylation pattern during aging. The authors identified 353 and 71 CpG sites, respectively [40,41], which were differentially methylated during aging and, therefore, can serve as reliable age predictors in human tissues. In fact, this “epigenetic clock” displays a robust correlation to age (r = 0.96 and r = 0.91, respectively) with minor deviations from the calendar age of analyzed individuals (3.6 and 4.9 years, respectively). In a comparative study evaluating the robustness of biological age predictors (i.e., epigenetic clock, telomere length, composite biomarker predictors, as well as transcriptome-, proteome- and metabolome-based predictors), the epigenetic clock was suggested to be the most reliable readout. However, further confirmatory studies will be needed to additionally evaluate the predictive values of these biological hallmarks of aging [42].

#### 2.1.4. ATP-Dependent Chromatin Remodeling

The chromatin is organized in a highly compact structure and in order to initiate cellular processes such as transcription, DNA replication, and DNA damage repair, it must be remodeled to enable the accessibility for required factors to the DNA. The reorganization of chromatin structures is facilitated by ATP-dependent chromatin remodeling complexes and results in activation or repression of transcription. These remodelers are multi-subunit complexes containing a highly conserved ATPase subunit which belongs to the superfamily II helicase-related proteins [43]. Based on their ATPase-flanking domains, these complexes are categorized into four major subfamilies: switch/sucrose non-fermentable (SWI/SNF), chromodomain helicase DNA-binding (CHD), INO80, and imitation switch (ISWI). They utilize ATP hydrolysis to disrupt interactions between DNA and histones leading, for instance, to nucleosome sliding/repositioning, nucleosome eviction and histone replacement/incorporation [44]. Despite the high complexity and redundance of several remodeling subunits, the understanding of chromatin remodelers and aging has increased over the last years. 

Recent data implicate that the two mutually exclusive catalytic ATPase subunits of the SWI/SNF complex, BRM (SMARCA2), or BRG1 (SMARCA4), are involved in telomere maintenance. BRG1 was identified as a negative modulator of the human telomerase reverse transcriptase (hTERT), an enzyme maintaining telomere ends. It was discovered that BRG1 levels are negatively correlated with hTERT in human cervical cancer cells and that BRG1 knockdown promoted hTERT transcription levels [45]. In a later study by these authors, it was shown in human fibroblasts and cervical cancer cells in vitro that BRM is required for the transcription of the telomere-binding proteins TRF1 and TRF2, which are essential to maintain telomere length and structure. This study provided novel insights into the longevity-associated functions of SWI/SNF by maintaining telomere structure and functionality [46]. In *C. elegans*, SWI/SNF and DAF16/FOXO co-localize at DAF16/FOXO target promoters to induce transcription of genes associated with stress resistance and longevity [47]. Mutations in LET-418, the *C. elegans* homolog of CHD3/CHD4, increased lifespan and stress resistance and this phenotype depends on DAF16/FOXO activity [48].

Similar to SWI/SNF, INO80 is essential for murine telomere maintenance and its deletion resulted in cellular proliferative defects and activation of p21-dependent cellular senescence [49].

ISW2, the catalytic component of the ISW2 complex, was described as a regulator of aging in *S. cerevisiae* since its deletion increased yeast replicative lifespan. By de-repressing stress response genes, the deletion of ISW2 contributed to promoting stress resistance pathways [50]. Subsequent research in *C. elegans* provided evidence that *isw1* gene expression is upregulated in response to multiple stressors changing histone levels. Accordingly, deletion of ISW1 resulted in reduced lifespan in the P0 and F1 generation, suggesting that ISW1 regulates longevity in worms [51].

#### 2.1.5. Histone Modifications

N-terminal tails protruding from the four core histones can be subjected to post-translational modifications. Depending on the histone, residue and type of modification (e.g., methylation, acetylation, phosphorylation and ubiquitination), these histone modifications can activate or repress transcription [52]. Importantly, the imbalance of repressing and activating histone modifications can change gene expression programs and, as a consequence, promote aging-associated transcriptome-wide changes.

##### Histone Methylation

While DNA methylation is highly associated with transcriptional repression, histone methylation can have both activating and repressing effects on gene expression. Previous studies imply that methylation of H3K4, H3K36, and H3K79 promotes transcription and that H3K9, H3K27, and H4K20 negatively affect transcription levels [53].

In *C. elegans*, the trimethylation of lysine 4 of histone H3 (H3K4me3) was demonstrated to negatively affect longevity. In fact, the loss of the H3K4me3 methyltransferase SET2 as well as WDR5 and ASH2 promoted survival, while the depletion of the H3K4 demethylase RBR2 showed the opposite effect [54]. This finding was supported in a study in *Drosophila* where the loss of *Lid*, the ortholog of RBR2, reduced life span in male flies [55]. Notably, 30% of H3K4me3-occupied genes displayed a substantial aging-related deregulation [56]. In agreement with those studies, H3K4me3 levels declined during aging in yeast and the authors described that its loss was linked to the induction of various aging-associated genes [57].

A survival screen *in S. cerevisiae* revealed that a deficiency in H3K36 methylation decreased life span. Accordingly, deleting the H3K36me2/3 demethylase RPH1 increased H3K36me3 levels and extended survival in yeast [58]. The decline in H3K36me3 occupancy during aging was confirmed in *C. elegans* and was associated with a transcriptome-wide age-dependent change of gene expression patterns. When inactivating the methyltransferase MET1 in *Drosophila*, animals displayed lower H3K36me3 levels as well as reduced lifespan [59]. Recently, the importance of H3K36me2 was demonstrated in aging *C. elegans*. When deleting the H3K36 dimethyltransferase SET18, lifespan was extended in a DAF16-dependent pattern [60]. Despite their distinct functions, these findings support a role of di- and trimethylation of H3K36 in longevity.

The trimethylation on lysine 27 of histone H3 (H3K27me3) is mediated by the Polycomb Repressive Complex 2 (PRC2) and removed by Ubiquitously Transcribed TPR on X 1 (UTX1). H3K27me3 negatively affects gene transcription and was shown, together with UTX1, to be reduced during aging in *C. elegans*. By depleting the demethylase UTX1 and thereby increasing H3K27me3 levels, the lifespan was increased [61]. Surprisingly, other studies revealed that increased H3K27me3 abundance was detected during aging in other species. For instance, depleting homologs of PRC2 members in flies resulted in increased longevity [62,63]. Moreover, quiescent mouse muscle stem cells and killifish brain tissues displayed an increase in H3K27me3 during aging [8,64].

The relevance of H3K9me3 was implicated in *Drosophila* when the deletion of KDM4A, a H3K9me3 demethylase, resulted in impaired wing extension and decreased lifespan in male flies [65]. Accordingly, among the factors downregulated after KDM4A loss, was the male sex-determination gene, as well as several genes associated with longevity [65]. In addition, decreased expression of the H3K9me3 methyltransferase *SUV39H1* was found during aging of human and murine hematopoietic stem cells [66].

Besides modifications of histone 3, also the trimethylation of lysine 20 on histone 4 (H4K20me3) has been linked to aging. When kidney and liver samples were collected from rats aged 10, 30, 300, and 450 days, a significant enrichment of H4K20me3 was detected in animals older than 30 days. In contrast, the amounts of mono- and dimethylated H3K20 did not change during aging processes [67]. In addition, it was described that senescent primary human IMR90 fibroblasts accumulated H4K20me3 in vitro [68].

##### Histone Acetylation

The addition of an acetyl group to the ε-amino group onto a lysine residue of a histone is believed to neutralize the positive charge of the lysine. As a result, the interaction between DNA and histone is weakened, thereby loosening chromatin structures and activating transcription. The addition and removal of acetyl groups is orchestrated by histone acetyltransferases (HATs) and histone deacetylases (HDACs), respectively.

When investigating histone modifications in livers isolated from 6, 15, and 30 month old rats, it was discovered that H3K9ac levels decreased with age [69]. In addition, the function of sirtuin 6 (SIRT6), a H3K9 deacetylase, has been addressed in several studies. In human fibroblasts in vitro, the depletion of SIRT6 resulted in abnormal telomere structures, which were similar to the situation observed in Werner syndrome, a premature aging disorder. The authors postulated that this finding is based on H3K9ac-related changes in chromatin states at telomeres [70]. In addition, SIRT6 appears to severely impact survival by regulating several essential biological functions. For instance, the global deletion of *Sirt6* in mice resulted in severe metabolic effects and an overall survival of only four weeks [71]. The observation that *Sirt6* deletion resulted in early lethality in mice was supported by another study. Here, it was discovered that inflammatory signaling pathways are targeted by SIRT6. It was shown that SIRT6 represses the expression of Nuclear Factor kappa B (NF-κB) target genes by deacetylating H3K9ac on NF-κB promoters [72]. In addition, lifespan of *Sirt6* knockout mice was described to be regulated via the insulin-like growth factor 1 (IGF1) pathway [73]. In agreement with these findings, it was recently demonstrated in a rat model that the overexpression of *Sirt6* suppresses senescence as well as apoptosis [74].

After the detection of H3K56ac in yeast [75], it was also discovered in mammals. Interestingly, the mutation of this lysine residue to glutamine (K56Q) or arginine (K56R) was associated with increased levels of spontaneous DNA damage and genotoxic stress. In addition, it was demonstrated that H3K56ac required the presence of the histone chaperone ASF1 and that it is mainly present during the S phase [76]. Besides deacetylating H3K9 as previously mentioned, SIRT6 has been described to deacetylate H3K56ac, thereby controlling DNA damage response and genomic stability [77].

The deacetylase SIRT7 positively regulates HAT1, augmenting the acetylation of H4K12 [78]. The function of SIRT7 lies in preserving the genome integrity and intestinal homeostasis [78]. Interestingly, SIRT7 was described as a regulator of aging since its loss caused aneuploidy and aging phenotypes in mice [78].

In addition, the H4K16 acetylation holds an important role in the regulation of telomere silencing, nucleosome assembly and maintaining chromatin structure [79,80]. The deacetylation of H4K16 is performed by SIRT1, whereas the supplementation of SIRT7 was able to prevent this event. A lower acetylation status was linked to defects in DNA repair and a senescent phenotype in mice [81].

The histone variant H2AX and its phosphorylation on serine 139 (γH2AX) have been tightly connected to aging since they depict an early event in the DNA damage response. Despite appearing in healthy brain areas, the cerebral cortex of senescent mice displayed a strong abundance of γH2AX at double strand breaks and repair sites [82]. In addition, the histone H3 threonine 11 phosphorylation (H3pT11) was described as an indicator for stress and aging in yeast and H3pT11 defective mutants displayed prolonged lifespan [83].

##### Histone Ubiquitination

Histone ubiquitination constitutes a post-translational modification, the transfer of a small regulatory protein, ubiquitin, to histone core members H2A and H2B. While the H2A ubiquitination mostly inhibits gene expression, H2B ubiquitination enhances transcriptional activity. H2B monoubiquitination is necessary for trimethylation of H3K4 and H3K79 and controls the binding of Cps35 with COMPASS complex, actively regulating transcription [84,85,86]. The age-dependent ubiquitination of H2A was originally found in *Drosophila*, and verified as evolutionarily conserved in humans [87]. In human glioma cells, the inhibition of the monoubiquitination H2Bub1 induced a cellular senescent phenotype [88].

#### 2.1.6. miRNAs

MicroRNAs (miRNAs), short and noncoding single-stranded RNAs (19-22 nucleotides), have become an emerging aging research interest over the last years. Through sequence-specific binding to their gene targets, miRNAs are able to repress translation or induce mRNA degradation [89,90]. Thereby, they exert a regulatory function in various cellular processes such as proliferation, differentiation and cell death [91]. Nearly 2000 miRNAs have been identified in humans which appear to be involved in the regulation of approximately 60% of all human genes [92,93]. An extensive overview on the individual functions of miRNAs affecting lifespan can be found elsewhere [90].

The most widely used model organism to evaluate the role of miRNAs in aging is *C. elegans*. One of the first discoveries in this field was that the miRNA *lin-4* targeting the transcription factor *lin-14* was not only required during the development, but also for aging processes in *C. elegans*. *Lin-4* overexpression or interfering with *lin-14* activity extended lifespan in a DAF16/HSF1-dependent manner [94]. These findings were supported by a study in *Drosophila* where the loss of mir-125 (a homolog of *lin-4*) reduced lifespan in male flies [95]. However, the function of this miRNA in humans remains unclear. 

While there is no to little knowledge regarding miRNA functions in aging across species, a recent study analyzed whole-blood samples from 5000 individuals and identified 127 miRNAs expressed in an age-related pattern (r = 0.70) [96]. In addition, several miRNAs have been implicated during the development or progression of aging-associated diseases as explained in Section 2.2.

### 2.2. Epigenetic Changes in Aging-Related Diseases

Epigenetic discoveries helped to lay the foundation for a deeper perception of multiple diseases. For instance, epigenetic events can contribute to the “hallmarks of cancer” (i.e., chromatin structure affecting cellular identity or methylation patterns leading to evasion from apoptosis), having led to a revised “hallmark” definition [97,98]. Here, we summarize the latest epigenetic discoveries in a selected range of medical conditions with a focus on certain cancer entities, inflammation, musculoskeletal disorders, neurodegenerative diseases, and nutritional diseases.

#### 2.2.1. Cancer

DNA methylation patterns and miRNAs can influence chromatin state regulation [99,100]. In cancer development, the degenerated cell may unrestrictedly proliferate as a consequence of DNA hypermethylation or deregulation of epigenetic modifiers. Methylation of the promotor region of the tumor suppressor genes, for instance *VHL*, was associated with angiogenesis, leading to an enhanced supply for the tumor environment. Cell death was shown to be impaired by epigenetic modification of apoptotic or cell cycle key players including CDKN2A, hypermethylation of which leads to a loss-of-function gene in numerous cancers [99,100,101]. Here, we focus on two entities to exemplarily demonstrate age-related shifts and deregulations associated with leukemia and colorectal cancer.

##### Leukemia

Distinct chromatin states can favor oncogene activation driving cells to the hallmarks of cancer [97,100]. For instance, a mutation or generated fusion-protein in the histone methyltransferase MLL or the histone acetyltransferase p300 can block regulatory regions in leukemia, driving malignant transformation [100,101,102,103,104,105,106,107,108]. The pivotal role of p300 in aging was demonstrated by Sen et al., who used 3000 shRNAs to silence well-known epigenetic proteins (*n* = 600) in order to identify candidates delaying replicative senescence. Upon treatment with shRNA directed against p300, the replicative lifespan of fibroblasts was substantially increased, accompanied by a reduced occurrence of telomere dysfunction-induced foci (TIFs) [109]. While the downregulation of p300 may delay cellular senescence, the downside of this targeting approach is that it appears to promote cancer. The importance of p300 for leukemogenesis was demonstrated by Cheng et al. The authors used a bone marrow transplantation mouse model for acute leukemia (donors: NHD13 p300^flox/flox^) in which the deletion of p300 led to reduced survival after acceleration of leukemogenesis. An enhanced self-renewal of hematopoietic stem/progenitor cells, combined with a decreased apoptosis rate and upregulation of cytokine receptor genes in p300-deficient cells drove leukemogenesis, identifying p300 as tumor suppressor [110]. In contrast, in a study where the suppression of p300 has been induced pharmacologically, the clonogenic growth of human leukemic cells was impaired [111]. Thus, these effects of p300 suggest a beneficial effect of reducing p300 activity during aging by enhancing cellular lifespan. However, depending of the biological context, decreasing p300 levels and activity may promote tumor development and progression due to its potential role as a tumor suppressor. 

Similarly, a dual function was described for the histone methyltransferase and Polycomb Repressive Complex 2 component EZH2, which has been proposed both as a tumor-suppressor and oncogene [112]. A gain-of-function EZH2 mutation characterized by an aberrant H3K27me3 occupancy blocked B cell development and was found in several lymphomas [112,113], whereas a loss-of-function of EZH2 is common in myelodysplastic syndromes [114]. Comparing human and murine methylation data sets, it was demonstrated that the pattern of overall DNA methylation with age is highly conserved between both species and that CpG islands, which are hypermethylated in an age-dependent manner associate with EZH2 [115]. 

Leukemogenesis has been found to be linked to further aberrant fusion proteins comprising RUNX1, RARα and CBFB [100,116]. In general, a feature of leukemia is the profound increase in H3K27me3 which can be attributed to GSK126, an inhibitor of EZH2 [113]. Subsequently, EZH2 inhibitors were discussed as therapeutic options in AML patients [117]. 

##### Colorectal Cancer

As one of the leading causes of cancer-related deaths worldwide, colorectal cancer can be driven by both genetic and epigenetic alterations within epithelial cells. An overall enhanced DNA methylation pattern has been found not only in inflammatory but also cancerous epithelial tissue [118,119]. In fact, this increase in methylation was associated with higher DNA methyltransferase 1 levels. Guo et al. characterized human colon cancer cell lines (SW480, LoVo, and HT29) as well as publicly available data based on colorectal adenocarcinoma samples to demonstrate a high *DNMT1* expression levels, accompanied by DNA hypermethylation. Interestingly, the Wnt signaling pathway was activated in these samples, indicating a positive regulation effect of Wnt signaling members on *DNMT1* expression while Wnt signaling inhibition downregulated DNMT1 [120]. The strong association between Wnt signaling and DNMT1 has been further elucidated using mass spectrometry-based proteomics. In colorectal cancer cells, a direct protein-protein interaction between β-catenin, the central effector of canonical Wnt signaling and DNMT1 was identified. DNMT reduction via siRNA reduced β-catenin signaling by decreased methylation at several CpG loci confirming the tight interdependence between the two tumor-promoting factors DNMT1 and β-catenin [121]. Besides epithelial cells, the function of peripheral blood mononuclear cells (PBMCs) was evaluated in colorectal cancer. It was demonstrated in PBMCs from 2453 European blood samples that DMNT1 levels gradually declined with aging until the age of 64 years [122,123,124,125]. Since the risk for colorectal cancer increases with aging, an in-depth analysis of the effect of DNMT1 reduction appears to be necessary. Surprisingly, Yung et al. showed an increased DNA methylation pattern via SssI methylase assays in several tissues along with reduced signs of senescence in heterozygous *Dnmt* null mice. In contrast, Laird et al. reduced the DNMT1 activity genetically and pharmacologically, resulting in a colonic hypomethylation and reduced number of intestinal neoplasia in *Apc*^Min^ mice [126,127]. Deductively, the role of DNMT1 in the aging intestine is still ambiguous and needs further in-depth characterization.

Colorectal inflammation, as one of the major risk factors of colorectal cancer, was found to be tightly connected to p16INK4a methylation: Wang et al. detected hypermethylation in the promoter region of p16 in human colonoscopic biopsies of rectal inflammatory mucosa, while Hsieh and colleagues suggested the hypermethylation of the p16INK4a promoter region to occur early during the neoplastic progression of ulcerative colitis in colectomy specimens [122,124]. Consequently, attempts have been made to identify molecular subgroups according to their methylation status in distinct gene sets. Accordingly, a CpG island methylator phenotype (CIMP) was identified for diagnostic and therapeutic stratification [119,120,121,122,123,124,125,126,127,128,129]. Of particular interest is the hypermethylation of the hMLH1 promoter, which was found in half of the patients with microsatellite instability and was associated with drug resistance [130,131]. These findings increase the understanding of epigenetic changes in colorectal carcinoma. The number of disparate critical drivers in colorectal cancer progressively leads to a “multi-gene, multi-drug” therapeutic strategy [132].

#### 2.2.2. Inflammation

It is broadly accepted that inflammation is a common event during aging, referred to as “inflamm-aging” [133]. This process is a underlying condition of several diseases such as sarcopenia, osteoarthritis, and cancer [134]. A hallmark of these processes is an increase in Tumor Necrosis Factor alpha (TNFα) levels. DNA methylation and histone acetylation modify the promoter region of TNFα [135]. The TNFα gene itself does not contain a classical CpG island, however, its promoter and first exon were described to be rich in CpG sequences [135]. Accordingly, methylation on these gene regions has been described to negatively regulate TNFα expression levels [136]. Wang et al. demonstrated in porcine spleens by bisulfite sequencing PCR and qPCR that the TNFα promoter region was increasingly methylated with age, correlating with decreased mRNA expression [137].

Similarly, NF-κB mediates acute, as well as chronic inflammation, and is proposed as one of the key regulators of aging. Via its transcriptional activity, the NF-κB family induces the expression of cytokines and genes associated with apoptosis and senescence as described elsewhere [138]. NF-κB levels can be regulated by various epigenetic mechanisms including the acetylation of histone H3 via the H3 lysine 4 methyltransferase SET7/9 [139] which represents a potential targeting strategy [136]. The link to aging has been validated in the skin where C57BL/6 mice exposed to UVB light for 16 days displayed accelerated aging of the skin via NF-κB activation through the mTORC2 pathway. The post-translational modification of the p65 member of NF-κB at Ser536 enhanced NF-κB activity via increased DNA binding activity. The same phenomenon was detected in physiological aged skin of these mice, demonstrating an accelerated inflammatory status in physiological as pathological (skin) aging [140,141]. 

Another epigenetic modification, which has been demonstrated to regulate NF-κB activity during inflammation, is the monoubiquitination at lysine 120 of histone H2B (H2Bub1). This monoubiquitination is performed by the RNF20/RNF40 E3 ligase complex and leads to increased chromatin accessibility. This results in eased passage of RNA Polymerase II and highly active transcriptional elongation [142]. Recently, it has been demonstrated that the monoubiquitination of histone H2B regulates NF-κB signaling in intestinal inflammation. However, the function of H2Bub1 in animal models for colitis remains inconclusive [143,144]. 

In general, the transformation from chronic inflammation to cancer can be promoted via DNA methylation, histone modifications, chromatin remodeling and noncoding RNA regulation, upon which the most important pathways are NF-κB- and STAT3-related [122,145]. The phosphorylation of Tyr705, Ser727, and Ser727 are known to positively activate transcriptional activity of STAT3 [145,146]. One downstream target of this signaling pathway is interleukin-6, which has been shown to be repressed via treatment with the DNMT1 inhibitor 5-azadeoxycytidine (5-AzaC) [122]. MicroRNAs are further key regulators of inflammatory responses and inflamed tissues are characterized by downregulation of TET gene expression due to the upregulation of TET-targeting miRNAs (e.g., MiR20a, MiR26B, MiR29C, Let-7 microRNA) [147,148].

Interestingly, the “epigenetic clock” concept of Horvath, has been demonstrated to be very accurate when methylation levels of CpG sites from white blood cells, the central regulators of immune response, were used [149,150,151]. These methylation levels were even able to predict mortality [152]. Despite a loss of T-cell diversity in old age, an exhausted/senescent CD8^+^ T cell population increases with age, possibly giving rise to associated diseases [153,154,155,156,157].

Excessive inflammatory response, immunosenescence, and autoimmunity outline the other detrimental side of the inflammatory spectrum. The identification of hypomethylated apoptosis-related genes in naïve CD4^+^ T cells led to the definition of an evolving autoimmune epigenotype [158,159,160]. Accordingly, in chronic nonbacterial osteomyelitis, a reduced expression of immunoregulatory cytokines (IL-10, IL-19) was centrally involved. The authors demonstrated in monocytes from chronic recurrent multifocal osteomyelitis patients that an altered SP1 activation negatively affected *IL10* and *IL19* expression. Mechanistically, the reduced phosphorylation of histone 3 serine 10 (H3S10P) and impaired SP1 phosphorylation at the *IL10* and *IL19* promoter regions impaired *IL10* expression. This causes an imbalance towards proinflammatory cytokines (compared to anti-inflammatory IL-10 and IL-19), leading to inflammatory bone loss [161]. Similar reductions of H3S1p levels have been identified in hippocampi of aged mice by Wu et al. [162], which were associated with the inflammation-related decline in spatial learning and memory.

#### 2.2.3. Osteoporosis

Osteoporosis is tightly linked to aging via epigenetic changes in mesenchymal stem cells (MSCs) [163]. Physiologically, the Osterix promoter was shown to entail enriched levels of H3Ac/H3K4me3 and reduced levels of H3K9me3/H3K27me3, inducing the differentiation of MSCs into osteoblasts to mediate skeletal tissue homeostasis [163,164]. Among the key transcription factors for osteogenesis are HOX and RUNX2, both of which are hypermethylated in aged MSCs [165]. Bork and colleagues demonstrated in MSCs isolated from bone marrow aspirates from young and old human donors that long-term cell culture and regular aging result in similar epigenetic profiles. HOXA (2,5,6) and RUNX2, transcription factors involved in osteoblast differentiation, were the most prominent among genes hypermethylated during aging which leading to decreased gene expression and age-related bone loss [166].

To prevent the hypermethylation of *RUNX2*, the transcriptional activation of the methyltransferase DNMT1 can be inhibited via 5-AzaC treatment. As expected, 5-AzaC leads to a hypomethylation of genomic DNA resulting in increased expression of *RUNX2*, Osteocalcin (*OCN*) and Osterix (*OSX*). The beneficial effect of 5-AzaC was supported by Zhou et al. in cell culture experiments using MSCs. Besides a global reduction in methylation levels, an increase in osteogenic gene expression was detected as demonstrated via enhanced alkaline phosphatase (ALP) activity and, subsequently, aggrandized mineralization [165]. 

It was shown in human bone marrow stromal cells (BMSCs) that in osteoporosis the number of clonogenic BMSCs was reduced, corresponding to decreased levels of *Tet1* and *Tet2*, factors, which are able to erase DNA methylation. During normal osteogenesis, TET1 and TET2 levels were enhanced with an increased binding to the Osterix promoter [163,167]. Yang et al. detected an osteopenic phenotype and decreased *Runx2* expression in *Tet1*^−/−^*Prx1^cre^Tet2^fl/fl^* mice. After the authors demonstrated that the use of siRNAs against TET1 and TET2 led to reduced stem cell properties in bone marrow MSCs (BMMSCs), they found via RNA-seq and qPCR in their mouse model that miRNAs targeting *Runx2* gene expression were significantly higher in the knockout mice and a treatment with mimics of these miRNAs increased *Runx2* expression in BMMSCs. The authors used chromatin immunoprecipitation (ChIP)-qPCR to demonstrate that TET1 and TET2 directly bind to the CpG island of the *P2rX7* promoter, a gene which has been linked to exosome release in earlier studies. After a depletion of *Tet1* and *Tet2*, the subsequent methylation led to impaired self-renewal and differentiation potential on the stem cell level in the bone marrow, thus leading to an osteopenic phenotype [168]. Rising evidence shows a broad involvement of several miRNAs in osteoporosis, such as miR-297a-5p, miR-297b-5p, and miR-297c-5p. These are accumulating intracellularly, inhibiting *RUNX2* expression, and thereby promoting an osteoporotic phenotype. This TET/P2rX7/RUNX2 cascade may serve as a target for novel therapeutic approaches [168,169]. Again, the link of Tet1/2 to aging has been made by Gontier et al. in the mouse brain, where a *Tet2* reduction was detected in the hippocampi of aged mice, and the application of high-titer lentivirus encoding for Tet2 shRNA in young adult mice caused deficits in short-term and long-term learning [170].

Mechanistically, an imbalance between histone modifications of osteogenic and adipogenic genes was proposed as an underlying mechanism of the development of musculoskeletal diseases. For instance, HDAC3 promotes osteogenesis and inhibits lipogenesis, while EZH2 and HDAC6 show opposite effects [165,171]. In bone marrow aspirates from human adults, it was demonstrated via retroviral-mediated enforced *Ezh2* expression in MSCs that the differentiation potential into adipocytes was higher compared to vector control cells, along with reduced *Runx2* transcription. Subsequent siRNA-mediated EZH2 depletion led to enhanced *RUNX2* expression. Using ChIP-qPCR, the authors demonstrated that enforced *EZH2* expression in MSCs resulted in increased H3K27me3 on transcriptional start sites of *RUNX2*, leading to a suppression of osteogenesis and marking EZH2 as positive regulator of adipogenesis and negative regulator of osteogenesis [171,172,173,174,175]. In another study, age-related bone loss was found linked to an increase in EZH2. In osteoporotic mice, Jing et al. detected via ChIP-qPCR that EZH2 was enriched at promoters of Wnt pathway members in BMSCs and a knockdown of EZH2 decreased H3K27me3 occupancy on these factors enhancing the *Runx2* and *Osterix* expression and subsequently osteogenic differentiation. Accordingly, the authors suggested the H3K27me3 inhibitor DZNep as a potential therapeutic substance for anti-osteoporotic treatment [172].

This translation of epigenetic discoveries into the clinic has already been established for several years. In fact, bisphosphonates (increasing MiR191c-5p and miR-497-5p) and monoclonal antibodies, such as denosumab, regulate—among others—DNA methyltransferases, histone acetylases, deacetalyses, and other key factors associated with detrimental epigenetic alterations [173,174,175].

#### 2.2.4. Neurodegenerative Diseases

Neurodegenerative diseases are tightly linked to the process of aging. Twin-studies demonstrated that accelerated epigenetic age, i.e., a higher methylation level, was linked to the development and progression of neurodegenerative diseases [176,177]. Alzheimer’s disease (AD) and Parkinson’s disease (PD) depict two distinct forms of neurodegenerative diseases that have been found associated with epigenetic modifications [178]. 

##### Alzheimer’s Disease

In our aging society, Alzheimer is displays a major health burden and cellular senescence was suggested as a key regulator controlling the shift from physiological aging into neurodegeneration [179]. Interestingly, senescent cells show a decrease in repressive heterochromatin marks, including H3K9me3, H3K27me3, and H4K20me3, leading to altered transcription patterns. In particular, the H4K16ac occupancy was reduced in regulatory regions of genes linked to aging in AD patients. Nativio et al. found in human postmortem brain samples from the temporal lobe via ChIP-seq analysis that H4K16ac levels increased in elderly healthy, but not aged AD patients. In particular, the reduced acetylation in AD patients was prominent on *HIC1* (p53-mediated DNA-damage response) and Wnt pathway members (synaptic transmission and plasticity). A protective effect of H4K16ac against neurodegenerative diseases has been suggested and therapeutically explored using a miR-149-5p inhibitor to increase H4K16ac levels in the AD in vitro model 293/APPsw, resulting in reduced β-amyloid formation, potentially attenuating AD progression [180,181,182]. Another gene of interest is the silencing transcription factor REST, promoting cellular cell death in AD and protecting neurons against oxidative stress and β-amyloid toxicity. Notably, it was demonstrated that physiological aged human brains showed increased REST mRNA and protein levels compared to AD patients. While a conditional deletion of *Rest* in mouse brains (*Nestin-Cre:REST^lx/lx^*) promoted age-related neurodegeneration, the neuroprotective effect of REST was confirmed by transgenic expression of human REST in *C. elegans*, reducing the sensitivity to oxidative stress and Aβ toxicity [183,184]. Summarizing, REST induction frequently appears in the (beneficial) aging process, presumably protecting the brain, while its decrease (as in AD) was associated with neurodegeneration [181,185]. In addition, Hou and co-workers demonstrated in mice that the loss of REST in AD is connected to abnormalities in miR124 signaling. In fact, *Rest* overexpression suppressed miR-124 while, consequently, the inhibition of miR-124 decreased *tau* aggregation and rescued memory deficits in 10-month-old P3001S mutant mice [186]. Moreover, 7mi-RNA was found to be significantly increased in the plasma of AD patients which was also true for hsa-miR-27a-3p in the cerebrospinal fluid, whereas the plasma levels of hsa-let-7d-5p and hsa-let-7-g-5p were discovered as potential biomarkers [187,188].

##### Parkinson’s Disease

In PD, the methylation by DNA methyltransferase 1 (DNMT1) as regulator of the alpha-synuclein (*SNCA*) gene is crucial. DNMT1 interacts with α-synuclein and is required for its localization in the cytoplasm, thereby reducing its effect on biophysical properties of the DNA [189,190]. However, as determined in a genome-wide association study in combination with rigid-body dockings simulation, nucleotide polymorphisms in the methylation region of the first intron of *SNCA*, which is required for the interaction with DNMT1, may influence the susceptibility to PD [191]. While overall a lower methylation rate was observed in diverse brain regions in PD patients, an enrichment of H3K27ac at enhancer regions was found at the *SNCA* locus in multiple brain regions associated with PD via human genome-scale enhancer identification via ChIP-seq. A possible targeting approach of this hyperacetylation was found by Mittal et al. who used human SK-N-MC neuroblastoma cells to detect compounds to decrease *SNCA* expression. Β2AR (β2-adrenoreceptor) agonists were found to modulate *SNCA* transcription through H3K27 deacetylation at its promoter and enhancer sites, promoting dopamine neuron health by reduced *SNCA* expression. The authors subsequently demonstrated in a nationwide longitudinal analysis that the most commonly used β2AR agonist, salbutamol, was associated with a reduced incidence rate of PD [192,193,194]. Moreover, it has been demonstrated that α-synuclein directly binds histones and inhibits the acetylation of histone H3 via the SIRT2 deacetylase, leading to decreased H3K9 acetylation as found in postmortem Parkinson patients’ primary motor cortex [195,196,197]. The central role of SIRT2 has been demonstrated by Esteves and colleagues in transmitochondrial cybrids, who demonstrated that SIRT2 increases microtubule instability via α-tubulin deacetylation and *tau* hyperphosphorylation. SIRT2 inhibition (via AK1), on the other side, improved intracellular trafficking [195]. Recently, a part of the SWI/SNF complex, SMARCA4, has been linked to age-related dopaminergic degeneration. Making use of gene co-expression analysis in human brain samples and *Drosophila* PD models, Sun et al. demonstrated that SMARCA4 was upregulated with aging and a down-regulation of SMARCA4 via siRNAs (drosophila: Brm) in dopaminergic neurons restored life span, indicating possible future diagnostic and therapeutic approaches based on SMRCA4 [198].

#### 2.2.5. Diet, Nutrition, and Type 2 Diabetes

Obesity is pathophysiologically associated with the development of type II diabetes [199,200]. Oxidative stress and inflammation, metabolic impairment and accelerated aging on both the micro- and macrocellular level contribute to the pathogenesis of metabolic diseases [201,202].

##### Dietary Restriction and Hunger

One of the earliest indications of a regulatory mechanism beyond sole genetics was deducted from the Dutch Hunger Winter families. In this observational study, an early-life (gestation) adverse environmental factor like famine (in the Netherlands and years of World War II 1944–1945) led to decreased methylation levels at distinct CpG islands, for instance on the Insulin-like Growth Factor II (*IGF2*) gene, influencing long-term metabolic health even six decades later [203,204]. In particular, the risk for obesity and type 2 diabetes was significantly higher when certain CpG sites (cg00574958, cg06500161) exhibited a high methylation status [205,206].

In general, nutritional influences were demonstrated to affect cellular longevity and carcinogenesis by telomerase and CDKN2A (p16) modulation: glucose restriction inhibited cellular senescence via chromatin remodeling and histone acetylation as well as methylation of the *CDKN2A* promoter, impairing E2F1 binding [205,206]. Consequently, caloric restriction builds a connection between aging and cancer: It has been indicated that caloric restriction increases lifespan via modifying the mammalian Target of Rapamycin (mTOR)-pathway, insulin/IGF-1 like signaling and sirtuins. Li and Tollefsbol investigated the effects of glucose restriction using lung fibroblasts in vitro and found that glucose restriction inhibited cellular senescence by measuring SA-βgal activity. Underlying *CDKN2A* mRNA did not accumulate in glucose-restricted medium compared to the normal glucose medium which was due to increased H3K9me3 (inactive chromatin marker) occupancy at the *CDKN2A* promoter. In contrast, active histone marks (H3 acetylation and H3K4me2) were detected to a lower extent at *CDKN2A* promoter region in glucose-restricted medium. Together, glucose deficiency led to a decrease of age-related histone modifications, subsequently, to a reduced incidence of aging-associated diseases [205,206,207]. 

On the molecular level, dietary restrictions can be mimicked by polyphenols, a large family of organic compounds naturally contained in fruits, vegetables, and cereals [208]. Interestingly, Sirtuin-1 and the senescence-associated secretory phenotype (SASP) are regulated by polyphenols. With molecular dynamics simulations and fragment-centric topographical binding interface mapping, Hou et al. demonstrated that resveratrol, a natural phenol, stabilized SIRT1/peptide interactions by forming a new binding pocket [209]. Since SIRT1 protein levels were increased after calorie restriction, mimetics such as resveratrol are of great interest in the treatment of aging-related diseases. Additional induction of autophagy and neutralization of free radicals resulted in further modulation of age-related detrimental mechanisms [210,211].

##### High Fat Diet and Obesity

Since aging and obesity have been demonstrated to be closely linked to each other, demographical changes will lead to an increased incidence of obesity in the future [212]. An increased variability in DNA methylation patterns was determined via observational studies in obesity and related phenotypes, indicating epigenetic dysregulation [213]. Histone modifications display the interface through which diet and stress affect organisms on a subcellular level [214]. Analyzing genome-wide methylation profiles in peripheral blood from obese and lean young humans, distinct CpG sites were associated with obesity and a prediction model was able to project adiposity with 70% confidence. In fact, obesity has been linked to differential methylation patterns and variability in CpG sites in proximity to certain genes, out of which *HIF3A*, *CPT1A*, *CD38*, and *PHGDH* were described to be most important [202,215].

However, the effects of diet on epigenetic patterns appear to be long-lasting and do not affect just one generation: A high-fat diet in embryonic developmental stages substantially affected later life [216]. The maternal diet during gestational and lactational period resulted in severe long-term effects. Zheng et al. demonstrated that a high-fat diet in maternal rats affected the p16 (Ink4a) protein and mRNA levels in the mammary gland of the offspring. Subsequently, ChIP revealed reduced H4 acetylation at the p16 promoter at the CpG-rich sites and enhanced recruitment of HDAC3 to the p16 promoter regulatory region in the mammary glands of the offspring after a maternal high-fat diet [214,217]. While the effects of a high-fat diet on crucial epigenetic promoter sites affecting Ink4a were investigated in detail, the effects of this diet on p21 (Cip1), another cyclin-dependent kinase inhibitor, appeared to be less crucial. Zhang et al. analyzed the effect of a high-fat diet in the liver of male rats. After obtaining a diet rich in fat for 9 weeks, the senescence marker p16(INK4a) was increased due to a decrease of H3K27me3 in the coding region of p16. Histologically, a hepatic steatosis and fat accumulation were observed. Contrary, the expression and protein level of p21 was decreased after a high-fat diet with no significant chromatin modifications of this gene [214,218]. Intriguingly, consequences of high-fat diets can even be detected after a short time (5 days) overfeeding diet in healthy young men. When evaluating the DNA methylation status in 21 healthy young men’s skeletal muscle biopsies after a diet with 50% extra calories for five days, methylation changes were detected on 6508 genes in the skeletal muscle and could only be partly reversed after 6–8 weeks. This finding indicated a slow reversibility of methylation patterns which, in the long-term, may influence gene expression levels [219].

##### Diabetes

Besides all other aforementioned conditions, diabetes has been previously associated with the involvement of epigenetics. In fact, epigenetic deregulation has frequently been observed in the β-cells of the pancreatic islands which showed an impaired capacity to secrete insulin in diabetes patients [201]. Using pancreatic islets from human donors, it was demonstrated that, along with DNA methylation changes at their CpG sites, a high-glucose stimulation altered expression patterns of a subset of genes including *CHRNA5*, *GLRA1*, and *PDX1*. Generally, diabetic islets-associated genes showed an overall trend towards increased DNA methylation and a decreased expression of key genes linked with impaired insulin secretion. Pathogenetically, the DNA methylation status (especially CpG sites annotated to the transcription factor *PDX1*) can be directly elevated via high glucose and glycated hemoglobin (HbA1c) while differentially methylated regions in pancreatic islets (*PDX1*, *TCF7L2*, *ADCY5*) promote islet dysfunction [220,221]. Another underlying mechanism, supporting the “inflamm-aging” theory, has been unveiled by Sandovici et al. The authors analyzed pancreatic islets from young and old rats and found differential expression of genes regulating islet cell function, type 2 diabetes genes and inflammatory processes, while age-associated transcriptional differences were negatively correlated with promoter DNA methylation at these gene loci [222]. 

The multifactorial and complex polygenicity of diabetes mellitus suggests that the effect size of single genes and CpG sites is low. Among the genes that show decreased DNA methylation and increased gene expression rates in diabetic patients, *CDKN1A* (p21), *PDE7B*, and *SEPT19* are of predominant interest. It was discovered in pancreatic islands from diabetic and non-diabetic donors that *CDKN1A* was differentially expressed in diabetic islands. In fact, the impaired glucose-stimulated insulin secretion was associated with an increased expression and reduced DNA methylation pattern of *CDKN1A*. Via transfection of clonal β-cells with mock-methylated or methylated constructs, the promoter methylation of *CDKN1A* (p21) has been demonstrated to directly negative affect the transcriptional activity [223]. As a cyclin-dependent kinase inhibitor, *CDKN2A* (p16) regulates the cell-cycle progression to G1 and an overexpression led to decreased cell proliferation, an “aged” phenotype, influencing both islet and non-islet mechanisms driving the diabetic phenotype [223,224,225].

The majority of association studies has shown multiple gene loci for epigenetic regulation in these central mediators of type II diabetes, β-cells. Chen and colleagues characterized *Ezh2*^fl/fl^ mice and *Cdkn2a*^−/−^ mice to reveal that an increased *Ink4a* and *Arf* expression in β-cells was linked to a reduced proliferative capacity. While *Ezh2* levels declined throughout aging, INK4A levels increased. ChIP analysis uncovered that H3K27me3 occupancy regulating *Ink4a* and *Ezh2* was declining with age, while H3K4me3 and histone acetylation at the *Ink4a* locus ascended in older mice. The authors concluded from their study that EZH2-dependent histone methylation and repression of the *Ink4a*/*Arf* locus are required for β-cell expansion [223,226]. In a further study, the methylome of β cells was analyzed pancreatic islets from young and old mice using whole genome shotgun bisulfite sequencing (WGSBS). Overall, higher methylation rates (especially in CpGs with low methylation levels in youth), accompanied by a decline in replicative capacity, increased promoter methylation and decreased expression of cell cycle regulators were detected in “healthy” old β-cells. Intriguingly, this observation was associated with a functional improvement in aged murine and human islets [223,227].

In addition, a high relevance of immunoregulatory mechanisms has been detected in type I diabetes. Miao et al. compared blood cells from type I diabetes patients to healthy controls and performed ChIP to identify diabetic methylation profiles. Variations in H3K9ac levels were found at upstream regions of human leukocyte antigen (HLA)-DRB1 and HLA-DQB1 in monocytes from diabetic patients. An increase in H3K9ac levels at the same promoter regions of HLA-DQB1, which was hyperacetylated in type I diabetes patients was found after Interferon gamma (IFNγ) and TNFα stimulation. This suggests that methylation changes in the human leukocyte antigen (HLA) region could precede the development of type I diabetes [228,229]. HLA-DQB1 is among the most relevant HLAs for genetic predisposition since it was identified to be highly probable to contain methylation-variable positions, possibly indicating an early etiological state in the development of type I diabetes [230].

Upon further epigenetic regulatory elements in diabetes, micro-RNAs, such as miR-15a and miR-29b, were found to be downregulated in type 2 diabetes, whereas miR-27a and miR-320a were upregulated and might open the possibility for new diagnostic markers [187,231,232,233].

## 3. Conclusions and Perspectives

This review provides a comprehensive overview of the key role of epigenetic mechanisms in controlling aging as well as the development of aging-associated pathologies. Numerous studies focusing on aging-related molecular mechanisms using cell-based systems, experimental animal models, as well as primary tissues have profoundly contributed to the current knowledge on this topic.

**Figure 1:**
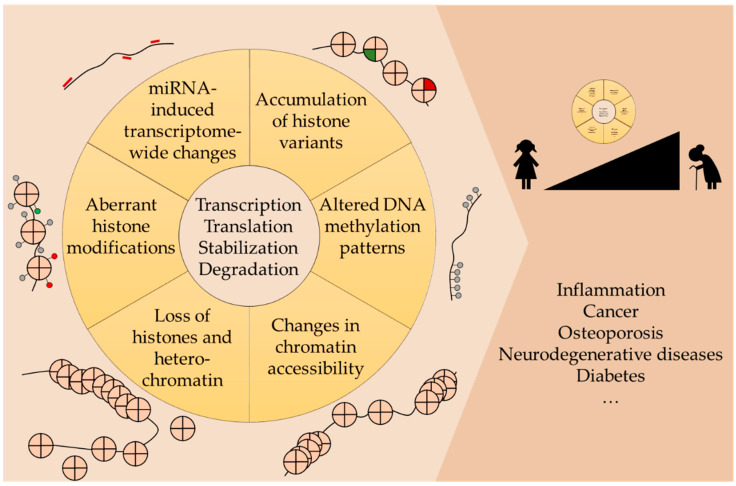
Epigenetics of aging and aging-related diseases. During aging, various epigenetic alterations occur including accumulation of histone variants, changes in chromatin accessibility mediated by chromatin remodeling complexes, loss of histones and heterochromatin, imbalance of activating/repressing histone modifications and aberrant expression/activity of miRNAs. These deregulations can affect transcription and, subsequently, translation, as well as the stabilization or degradation of molecular components. Consequently, these aberrant epigenetic processes can promote morbidities, which are frequently observed in the elderly populations, including inflammation, cancer, osteoporosis, neurodegenerative diseases, and diabetes.

It has been shown that distinct epigenetic mechanisms are enriched in aging organisms including the accumulation of histone variants as well as the loss of histones and heterochromatin (Figure 1). In addition, histones show aberrant post-translational modifications leading to the imbalance of activating and repressing modifications. Moreover, remodeling complexes modulate chromatin accessibility and there is an aberrant expression/activity of miRNAs. Together, these epigenetic deregulations contribute to aging-associated changes in gene transcription and, as a consequence, translation as well as the stabilization or degradation of molecular factors. While mechanisms underlying aging-related pathologies remain to be elucidated in detail, various studies demonstrate an epigenetic component. In fact, the aforementioned epigenetic modifications were shown to play essential roles in diseases including inflammation, cancer, osteoporosis, neurodegenerative diseases, and diabetes.

While the precise mechanisms and connections between several epigenetic changes and human pathologies are still poorly understood, state-of-the-art next generation sequencing methods will allow researchers to address remaining questions. For instance, chromatin accessibility using Assay for Transposase-Accessible Chromatin using sequencing (ATAC-seq) can be coupled to ChIP-seq as well as gene expression studies (mRNA-seq) using bulk mRNA or even analyzing single cells (scRNA-seq). In addition, advances in molecular biology and cell culture approaches (for instance Clustered Regularly Interspaced Short Palindromic Repeats (CRISPR)/Cas9) will be beneficial in clarifying aging-processes across species. 

An improved understanding of epigenetic mechanisms affecting longevity will be deciding crucial step towards the identification of new potential therapeutic targets. In fact, epigenetic drugs are of particular interest to the clinic due to their reversible and transient effect.

A limitation of manifold epigenetic studies, however, are the variations among single cells (both on an individual and tissue level), which occur with an even higher frequency in aged organisms [234,235]. This biologically relevant heterogeneity might be further investigated, understood and potentially deconstructed with the help of new technological approaches like single-cell genomics [236,237,238].

Together, characterizing molecular changes in different species during aging using state-of-the-art techniques will provide key insights into the relevance of epigenetics of aging and aging-associated diseases.

## Data Availability

Data sharing not applicable.
